# LIVING ALONE, HAVING CHILDREN AND FRIENDS NEARBY, AND DAILY FRUIT AND VEGETABLE CONSUMPTION

**DOI:** 10.1093/geroni/igz038.2379

**Published:** 2019-11-08

**Authors:** Yeon Jin Choi, Jennifer A Ailshire, Eileen Crimmins

**Affiliations:** 1 University of Southern California, Los Angeles, California, United States; 2 Davis School of Gerontology, University of Southern California, Los Angeles, California, United States

## Abstract

Fruit and vegetable consumption is associated with lower risk of chronic diseases and mortality. Despite the numerous health benefits, fruit and vegetable consumption of most older adults are below the daily recommendation. This paper aimed to investigate whether living alone and having children and friends nearby are associated with older adults’ daily fruit and vegetable consumption using a nationally representative sample of older Americans. Daily fruit and vegetable consumption was measured using (1) daily serving and (2) daily recommendation (2 or more servings for fruits; 3 or more servings for vegetables). Poisson and logistic regression models were estimated using the HRS Health Care and Nutrition Study. The sample included 6,915 community-dwelling older adults. Older adults who were living alone had lower fruit and vegetable consumption and less likely to meet daily recommendation for vegetables, compared to those who were living with someone. Having friends nearby was positively associated with the outcomes, while having children nearby was associated with meeting daily recommendation for vegetables only among older adults living alone. Based on the findings, older adults who are living alone and do not have children and friends nearby may be at the risk of poor nutrition due to low levels of social support. Provision of help with grocery shopping (e.g., transportation, Supplemental Nutrition Assistance Program) and meal preparation (e.g., home-delivered meals) as well as more social opportunities that can improve social support network and encourage healthy eating (e.g., congregate meals) may increase daily fruit and vegetable consumption of older adults.

